# RASAL3 predicts overall survival and CD8+ T lymphocyte infiltration in lung adenocarcinoma

**DOI:** 10.1111/jcmm.17625

**Published:** 2022-11-24

**Authors:** Mei Liang, Xiangzhi Meng, Boxuan Zhou, Yushun Gao

**Affiliations:** ^1^ Department of Thoracic Surgery, National Cancer Center/National Clinical Research Center for Cancer/Cancer Hospital Chinese Academy of Medical Sciences and Peking Union Medical College Beijing China

**Keywords:** CD8^+^ T lymphocyte infiltration, clinical characteristics, immunohistochemistry (IHC), lung adenocarcinoma, overall survival (OS), RASAL3, tissue microarray (TMA)

## Abstract

RAS‐activating protein‐like 3 (RASAL3) is a synaptic Ras GTPase‐activating protein (SynGAP) and a potential novel biomarker of CD8+ T cell infiltration in lung adenocarcinoma (LUAD). This study explored RASAL3 expression in LUAD, the prognostic impact of RASAL3 and the relationship with immune cell infiltration. RASAL3 expression in LUAD tissues was considerably low, with high RASAL3 expression associated with better overall survival, whereas the low expression was linked to advanced T, N, M classifications, TNM stage and lower grade. Furthermore, RASAL3 expression positively correlated with CD8+ T lymphocyte infiltration. In conclusion, RASAL3 expression is a potential prognostic and immunological biomarker of LUAD.

## 1 INTRODUCTION

Lung cancer is one of the most common causes of cancer‐related mortality globally, with lung adenocarcinoma (LUAD) accounting for 40% of all lung cancers.^1^, ^2^ Recently, immunotherapy has transformed the non‐small cell lung cancer (NSCLC) landscape.^3^, ^4^, ^5^ However, the effect of immunotherapy mainly depends on the immune response, which is greatly impacted by the tumour microenvironment.^6^, ^7^ CD8+ T cells are crucial in the tumour microenvironment, and highly infiltrated cells are useful in tumour prognosis involving LUAD.^8^, ^9^, ^10^ Nevertheless, CD8+ T cell infiltration in LUAD tumour microenvironment mechanism is unclear, so identifying novel biomarkers of CD8+ T cell infiltration may help explore the immune infiltration mechanisms in LUAD.

Ras proteins have pivotal functions in differentiation, proliferation and oncogenesis by acting as molecular switches in intracellular signalling pathways.^11^, ^12^ RASAL3 belongs to RASAL family, whose members all have C2, pleckstrin homology (PH) and RasGAP domains. As a Ras protein negative regulator, the active Ras (RAS‐GTP) can be converted to inactive Ras (RAS‐GDP) by RASAL3.^13^ Previous studies demonstrated that RASAL3 is a T‐lymphocyte‐specific Ras GTPase‐activating protein that negatively regulates Ras/MAPK pathway induced by T cell receptor (TCR) activation. Moreover, the number of naive T cells in RASAL3‐deficient mice was reduced as RASAL3 is necessary for its survival. Additionally, RASAL3 functions in NKT cell expansion in the liver by negatively regulating Ras/Erk signalling.^14^, ^15^

The tumour microenvironment and immune‐related mechanisms have a significant role in LUAD development and treatment, with tumour‐infiltrating lymphocytes (TILs) affecting prognosis and the efficiency of chemo and immunotherapies.^16^, ^17^, ^18^, ^19^ The tumour microenvironment promotes lung cancer progression and metastasis by regulating T cell recruitment. Indeed, about two‐thirds of inflammatory cells in NSCLC are lymphocytes, of which 80% are T cells, and lung cancer patients with high levels of infiltrated CD8+ and CD3+ T cells have a better OS.^20^, ^21^, ^22^, ^23^, ^24^, ^25^ This study explored the expression of RASAL3 and its prognostic effect in LUAD using TCGA and PrognoScan databases, as well as immunohistochemistry (IHC) based on a tissue microarray (TMA).

## 2 MATERIAL AND METHODS

### 2.1 Tissue microarray and immunohistochemistry

RASAL3 and CD8 expressions were explored using a tissue microarray (TMA) (Shanghai Outdo Biotech Co, Ltd.) involving 94 LUAD tissues and 82 paired normal lung tissues. All study participants did not receive neoadjuvant therapy or underwent surgery between May 2007 and December 2012. Bioethical approval and consent were taken before the tissue sample collection. LUAD diagnosis was confirmed by pathological evidence, with the tumour grade and clinical staging determined utilizing the 7th Joint Commission on Cancer (AJCC) TNM criteria. The National Cancer Center/Cancer Hospital of the Chinese Academy of Medical Sciences and the Peking Union Medical College Ethics Committee approved this research (IRB Approval No. Ncc2019c‐167).

The sections were mounted onto slides coated with 3‐aminopropyltriethoxysilane, then dried and dewaxed before antigen retrieval and incubation with antibodies against RASAL3 (orb317793, dilution: 1:500; Biobyt LLC., St Louis, MO 63132, USA) and CD8 (IR623, dilution 1:500; Dako). The slides were then incubated with HRP‐labelled anti‐rabbit IgG (Dako, Gloustrup) at room temperature for 45 minutes before staining with 3, 3‐diaminobenzidine (DAB), and haematoxylin. The staining was analysed using the EnVision+ detection System (Dako) and scored by multiplying the tissue staining intensity (0–3) and extent (0%–100%).

### 2.2 Survival analysis of RASAL3 in LUAD

TCGA and TMA cohorts were utilized to evaluate RASAL3 expression and prognosis correlation. Patients with other malignancies were excluded from TCGA cohort. The ‘survminer’ R package was used to calculate the cut‐off values for high and low expression. RASAL3 expression and LUAD prognosis were also investigated using PrognoScan database, a large publicly available cancer microarray dataset that can be used to explore the genes and clinical outcomes correlation.^26^ The hazard ratio (HR) and adjusted P values were evaluated, and the prognosis of patients with different RASAL3 expressions was compared by the Kaplan–Meier and log‐rank tests. Multivariate Cox regression analysis confirmed whether RASAL3 was an independent prognostic factor.

### 2.3 Clinical characteristics and RASAL3 expression in LUAD

TCGA and TMA cohorts were utilized to study the clinical characteristics and correlation with RASAL3 expression. The differential expression of RASAL3 in paired tumour and normal tissues was analysed. LUAD patients' age, sex, T, N, M and TNM stages were obtained from TCGA cohort to analyse RASAL3 expression in these different subgroups. UCSC Xena browser was used to download 20,530 genes of LUAD (TCGA‐LUAD) Level 3 RNA Seq V2 data, as well as the latest clinical and survival information data. Gender, age, T, N, TNM and grade were also collected and analysed in TMA cohort. In addition, CD8+ T lymphocyte expression was evaluated.

### 2.4 Immune cell infiltration

CIBERSORT method was employed to assess the immune cell proportions in LUAD. It is a tool for estimating the immune cell infiltration by deconvoluting the immune cell subtypes expression matrix relying on the linear support vector regression.^27^, ^28^ TCGA data portal (TCGA, Callisto, Welfare, Thibosport, Relative TSV) was utilized to download the immune cell fraction data^29^; then, the ‘Corrplot’ function in R software package was used to conduct the correlation analysis of RASAL3 expression and immune cell filtration. RASAL3 expression and correlation with immune cell infiltration including CD4 + T cells, CD8 + T cells, B cells, dendritic cells and macrophages were analysed using TIMER database. The immune infiltration abundance was estimated using 10,897 tumour samples containing 32 cancer types.^30^ The correlation in TIMER was adjusted by tumour purity in the left‐most panel.^31^ The correlation modules were represented as a scatter plot of expression of a specific gene pair, as well as Spearman correlations and statistical significance. The Y‐ and X‐axes represent RASAL3 expression and the corresponding gene markers in immune cell expression. LOG2‐RSEM was utilized to detect the gene expression level.

### 2.5 Statistical analysis

The data were reported as mean ± standard deviation (SD), and the differences were analysed using one‐way anova or Student's *t*‐test (number of variables =2). The Chi‐square test, log‐rank test, Kaplan–Meier analysis and Cox regression analysis were used to analyse the differences between categorical variables to estimate prognosis using the absolute values from the timer to determine the strength of the correlation.^32^ SPSS 23.0 software and R software version 3.4. were used for statistical analysis, and a *p* value <0.05 was considered significant.

## 3 RESULTS

### 3.1 RASAL3 expression is lower in LUAD tissues

**FIGURE 1:**
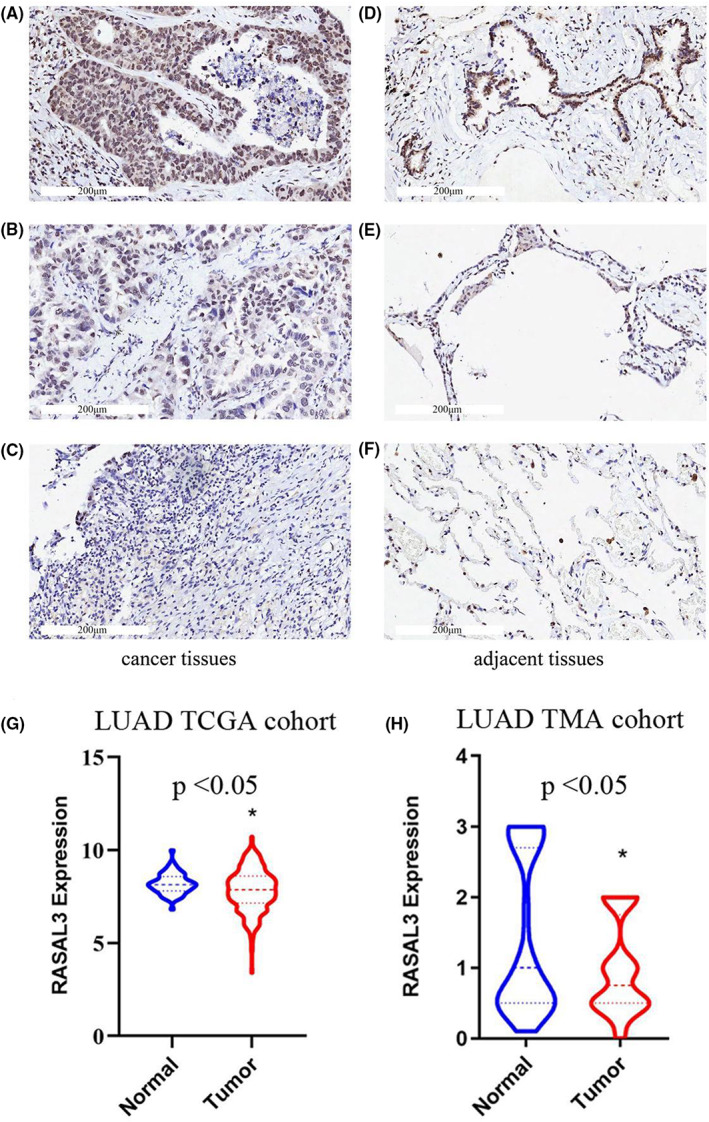
Immunohistochemical staining of RASAL3 in LUAD tissue samples and corresponding noncancer tissue samples. RASAL3 protein levels were down‐regulated in most LUAD tissues compared with the corresponding noncancer tissues in the TMA‐IHC results. The representative TMA‐IHC images of different staining intensities of RASAL3 were shown below (A–F). (A) Strong intensity of RASAL3 in LUAD tissue. (B) Moderate intensity of RASAL3 in LUAD tissue. (C) Weak intensity of RASAL3 in LUAD tissue. (D)Strong intensity of RASAL3 in corresponding noncancer tissue. (E) Moderate intensity of RASAL3 in corresponding noncancer tissue. (F) Weak intensity of RASAL3 in corresponding noncancer tissue. (G‐H) Differential expression of RASAL3 in normal tissues and LUAD by utilizing the TCGA and TMA cohorts. *p < 0.05

Five hundred and fifteen patients with LUAD were selected from the TCGA database. We first evaluated the differential expression of RASAL3 between 515 LUAD tissues and 59 adjacent tissues. LUAD tissues had lower RASAL3 expression than the normal tissues (Figure 1G). In TMA cohort, we analysed differential RASAL3 expression between 82 LUAD and adjacent tissues by IHC. IHC results showed that RASAL3 was mainly expressed in the cytoplasm and lower expression in LUAD tissues (Figure 1H). Different RASAL3 staining intensities in TMA cohort are displayed in Figure 1A–F.

### 3.2 High RASAL3 expression is associated with a better prognosis in LUAD

**FIGURE 2:**
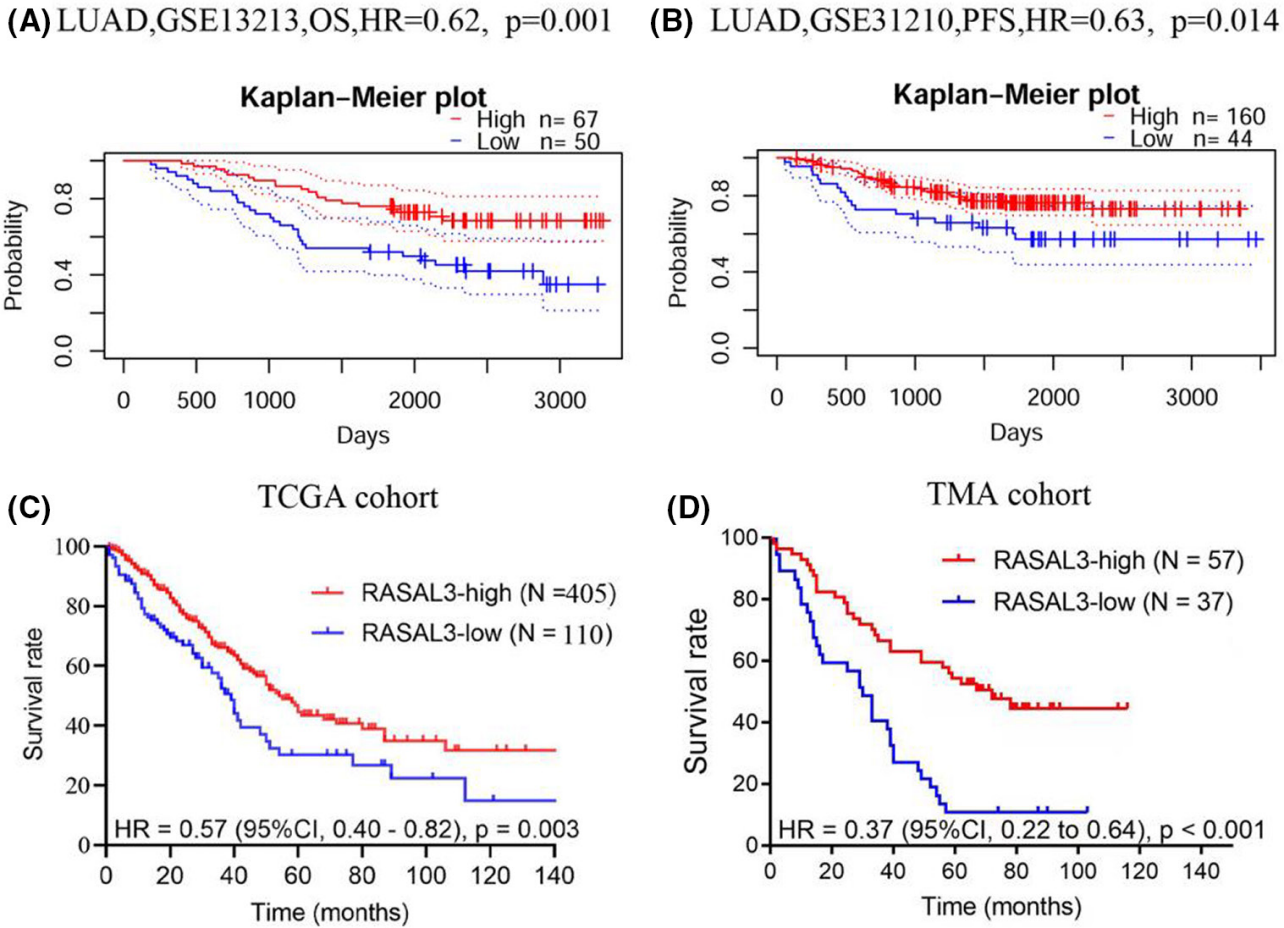
Relationship between RASAL3 expression and prognosis in LUAD analysed by utilizing PrognoScan database, TCGA cohort and TMA cohort. (A) In the GSE13213 cohort, high expression of RASAL3 predicted a better OS in LUAD (HR = 0.62, *p* = 0.001). (B) In the GSE31210 cohort, high expression of RASAL3 predicted a better PFS in LUAD (HR = 0.63, *p* = 0.014). (C) The Kaplan–Meier survival curve comparing the high and low expression of RASAL3 in TCGA cohort. (D) The Kaplan–Meier survival curve comparing the high and low expression of RASAL3 in the TMA cohort

The two PrognoScan cohorts (GSE13213 and GSE31210) results suggest that LUAD patients overexpressing RASAL3 had a better prognosis (Figure 2A and B). In addition, TCGA cohort with high RASAL3 expression had a better survival rate than those with low expression levels (Figure 2C). Furthermore, TMA‐based IHC to validate the RASAL3 expression prognostic value in 82 pairs of LUAD tissues and the adjacent tissues was consistent with PrognoScan database and TCGA cohort results (Figure 2D).

Cox multivariate regression analysis using TCGA and TMA cohorts revealed that RASAL3 expression and TNM stage were independent prognostic factors (Table 1); therefore, RASAL3 may be a prognostic biomarker for LUAD.

**TABLE 1:** Multivariable Cox regression analysis by utilizing the TCGA cohort and TMA cohort

variables	TCGA cohort			TMA cohort		
	HR	95%CI	*p* value	HR	95%CI	*p* value
Expression (low vs high)	0.636	0.457–0.885	0.007	0.496	0.249–0.986	0.046
Age (≤55 vs. > 55)	1.012	0.997–1.027	0.124	1.027	0.996–1.06	0.087
Gender(female vs. male)	0.924	0.682–1.253	0.612	0.938	0.513–1.718	0.837
TNM stage	1.632	1.418–1.878	<0.001	1.538	1.072–2.208	0.02

### 3.3 RASAL3 expression and correlation with clinicopathological characteristics in LUAD

**FIGURE 3:**
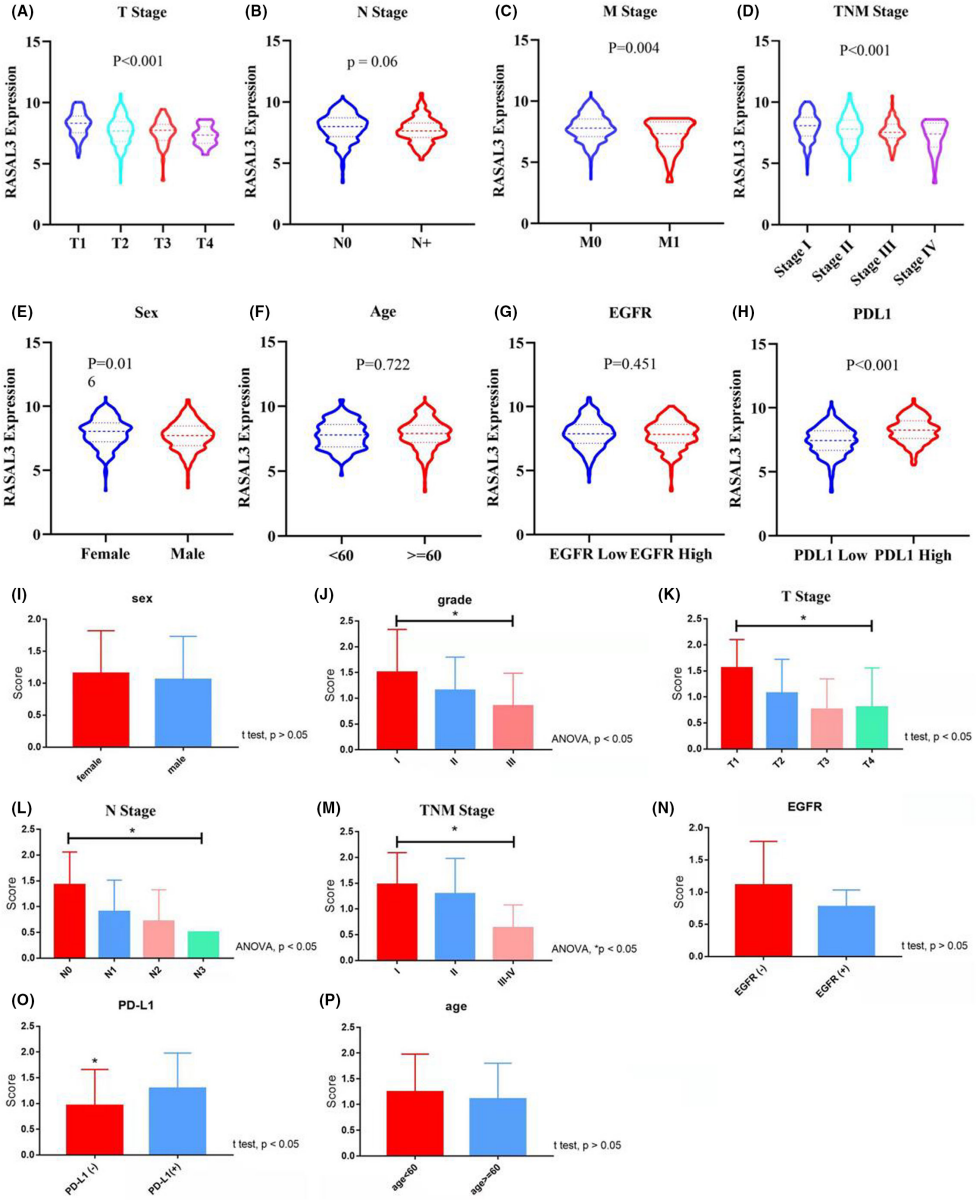
(A–H)Correlation analysis between RASAL3 expression and different clinical characteristics in LUAD via TCGA cohort. (I–P) The correlation analysis between RASAL3 expression and different clinicopathological characteristics in LUAD via TMA cohort. *p < 0.05

Low RASAL3 mRNA expression in TCGA cohort is linked to advanced T classification (*p* < 0.01), M classification (*p* = 0.004) and TNM staging (*p* < 0.001) but there was no significant difference between RASAL3 expression and age (*p* = 0.124) or gender (*p* = 0.612) (Figure 3A–H). RASAL3 protein expression in the TMA cohort was associated with T classification (*p* < 0.05), N classification (*p* < 0.05), TNM staging (*p* < 0.05), grade (*p* < 0.05) and PD‐L1(*p* < 0.05) (Figure 3I–P), implicating RASAL3 in LUAD progression.

### 3.4 Relationship between RASAL3 expression and immunomarker sets

**FIGURE 4:**
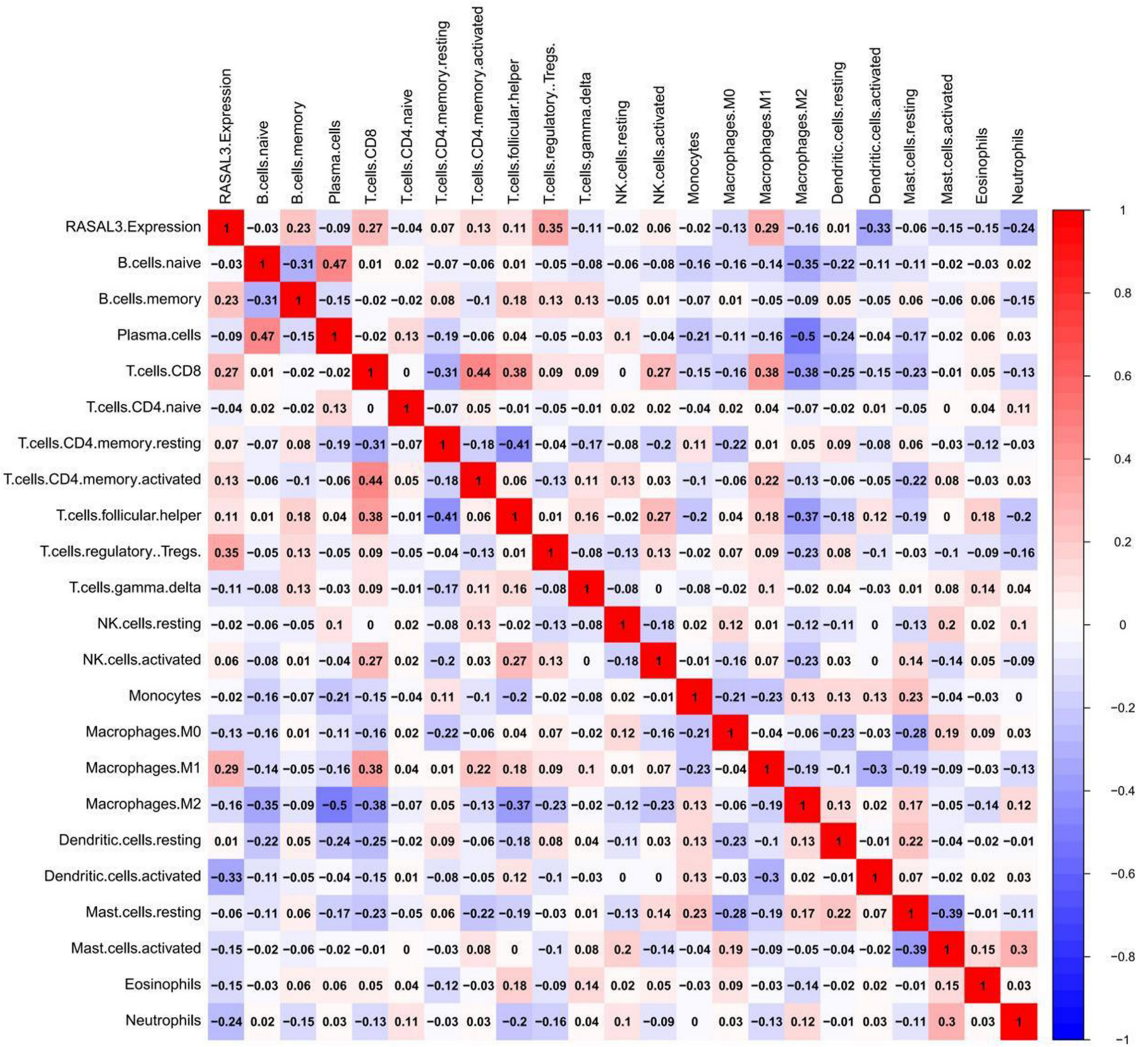
Relationship between RASAL3 expression and immune‐infiltrating cells by utilizing CIBERSORT algorithm: RASAL3 expression was positively correlated with CD8 + T cells, B cells, Tregs and M1 macrophages. RASAL3 expression was negatively correlated with dendritic cells, neutrophils and M2 macrophages

**FIGURE 5:**
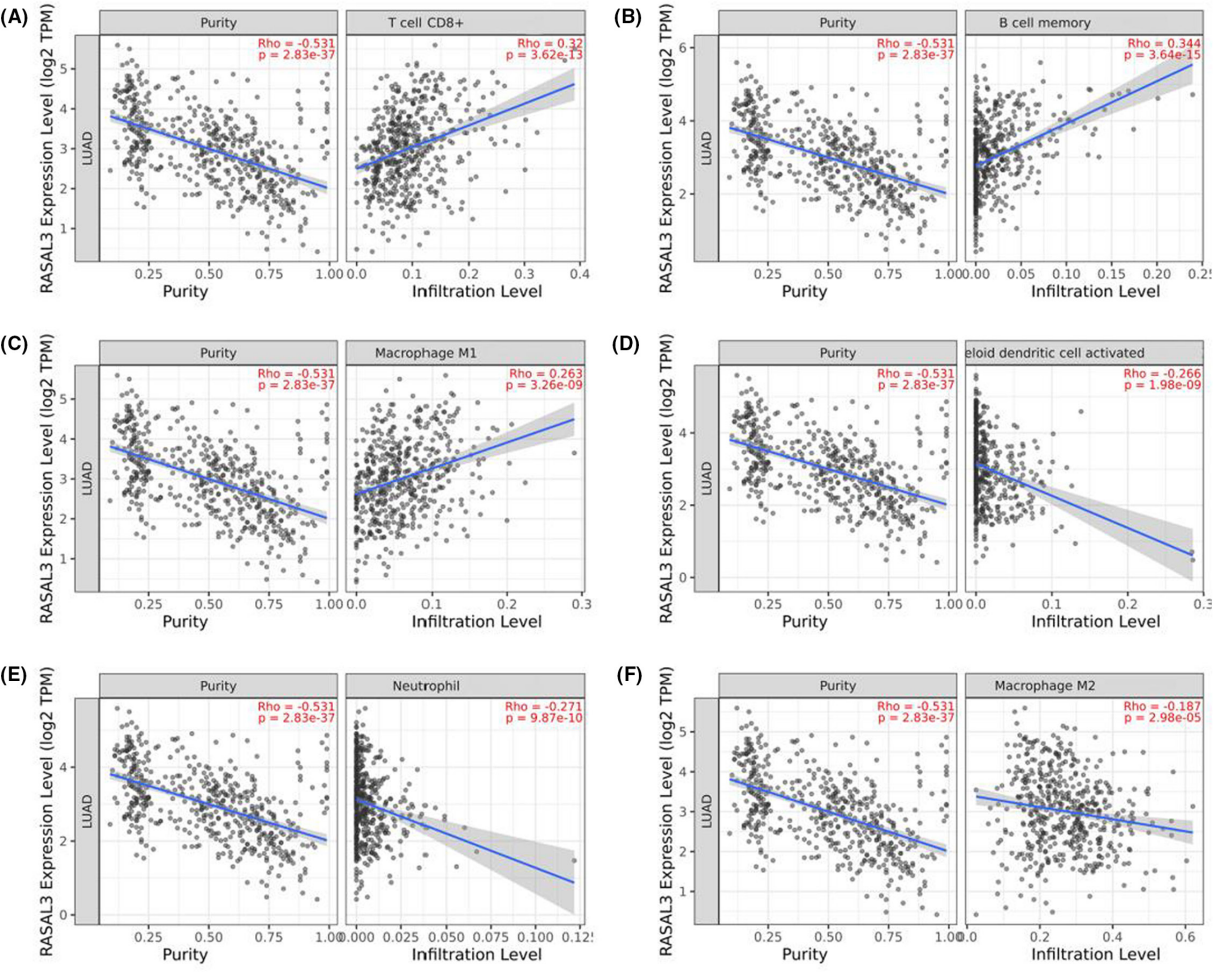
Relationship between RASAL3 expression and immune‐infiltrating cells by utilizing the TIMER database: RASAL3 expression was positively correlated with CD8 + T cells(A), B cells(B), M1 Macrophages(C). RASAL3 expression was negatively correlated with the infiltration of dendritic cells(D), neutrophils(E) and M2 macrophages(F)

**FIGURE 6:**
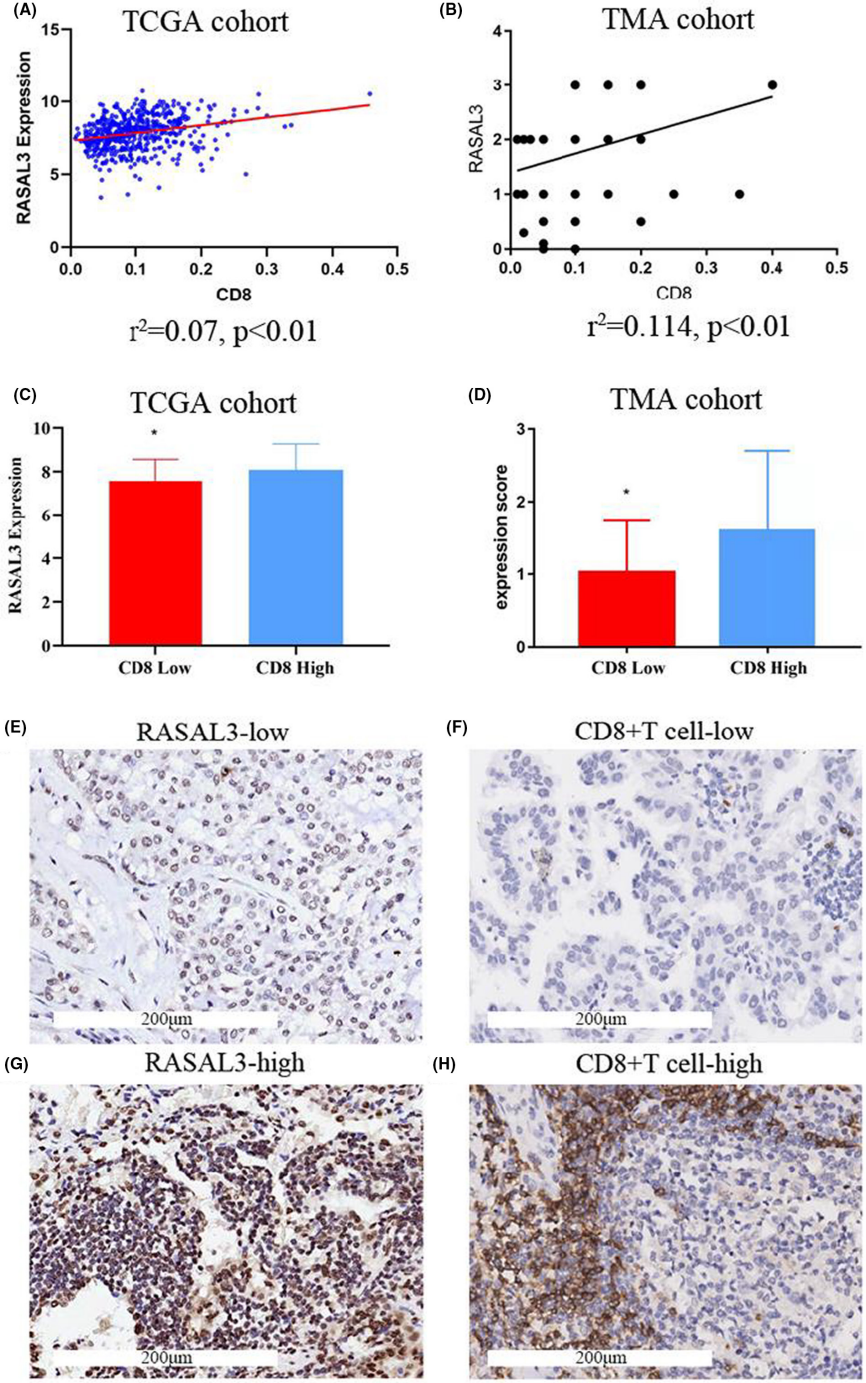
(A‐D)Relationship between RASAL3 expression and CD8+ tumour‐associated T lymphocyte infiltrations in LUAD. (A) The correlations between RASAL3 expression and CD8+ tumour‐associated T lymphocyte infiltration in TCGA cohort ((r2 = 0.27, *p* < 0.001). (B) The correlations between RASAL3 expression and CD8+ tumour‐associated T lymphocyte infiltration in the TMA cohorts (r2 = 0.114, *p* < 0.001). (C) The correlation between RASAL3 expression and CD8 expression in the TCGA cohort (*p* < 0.001). (D) The correlation between RASAL3 expression and CD8 expression in the TMA cohort (*p* < 0.001). (E‐H) The IHC staining of RASAL3 and CD8 in the same LUAD tissue samples. (E) Strong intensity of RASAL3 in LUAD tissue. (F) Weak intensity of RASAL3 in LUAD tissue. (G) Strong intensity of CD8+ in LUAD tissue. (H) Weak intensity of CD8+ in LUAD tissue. *p < 0.05

The correlation between RASAL3 mRNA expression and the immune markers was assessed by CIBERSORT algorithm, demonstrating that RASAL3 expression was positively correlated with the infiltration of CD8+ T cells, B cells, regulatory T cells (Tregs) and M1 macrophages (Figure 4). Meanwhile, RASAL3 expression was negatively correlated with the infiltration of dendritic cells, neutrophils and M2 macrophages. This relationship was further confirmed by analysing the correlation of RASAL3 expression with various immune cell infiltration levels (Figure 5A–F) using TIMER. Interestingly, CD8 mRNA expression was positively correlated with RASAL3 mRNA expression in TCGA cohort and TMA cohort (Figure 6A,B).

The correlation between RASAL3 expression and CD8+ tumour‐associated T lymphocyte infiltration was further validated by TMA‐based IHC (Figure 6 E‐H), confirming that RASAL3 expression was strongly positively correlated with CD8+ tumour‐associated T lymphocyte infiltration in line with the CIBERSORT algorithm, TIMER database and TCGA cohort results. CD8+ T lymphocyte infiltration levels were classified as low or high infiltration according to the median value revealing that mRNA and RASAL3 protein levels in the high infiltrating CD8+ group were considerably higher than in the low infiltrating group (Figure 6 C and, D), suggesting that RASAL3 may regulate T lymphocytes in LUAD. The high RASAL3 expression might increase the infiltration of CD8+ tumour‐associated T lymphocytes in LUAD.

## 4 DISCUSSION

Currently, the major treatment strategies for LUAD are surgery coupled with chemotherapy and targeted therapy. Indeed, immunotherapy plays an increasingly important role in tumour therapy of LUAD. The immune microenvironment in the tumour significantly affects treatment outcomes, with CD8+ T cells being important anti‐tumour effector cells.^33^, ^34^, ^35^, ^36^ Hence, the identification of key genes linked to CD8+ T cell infiltration may provide new ideas for studying mechanisms of tumour immunotherapy.

Previous studies have explored some prognostic biomarkers to predict prognosis in LUAD.^37^, ^38^ Interestingly, our study also elucidates that RASAL3 expression may play a role in LUAD and is a potential prognostic biomarker. We found that LUAD tissues had lower RASAL3 expression in comparison with the normal tissues. RASAL3 protein and mRNA expression were negatively correlated with T, N classifications, TNM stage and pathological grade in LUAD patients. In TMA cohort, the prognostic analysis also suggested that low RASAL3 expression in LUAD patients indicates a poor prognosis. The multivariate Cox regression analysis confirmed that RASAL3 was independently associated with prognosis. To our knowledge, this is the first study to explore the association between RASAL3 expression and clinical features in LUAD patients.

The family of RAS GTPase‐activating proteins has a crucial function in cell proliferation, function and development. RASAL1 gene, as a tumour suppressor gene, is normally expressed in many tissues but decreased in breast, liver, lung, oesophageal cancers, lymphoma, nasopharyngeal and oral squamous cell carcinomas.^32^, ^39^, ^40^ Additionally, genetically engineered RASAL2 knockout mice are prone to several sporadic tumours, including LUAD. RASAL2 inactivation could promote lung cancer cell migration and lung metastasis in nude mice by inducing epithelial‐mesenchymal transformation.^25^ However, RASAL3 has not been extensively studied in human solid cancer including LUAD. It was reported that RASAL3 could promote NKT cell expansion and function by negatively regulating RAS/ERK signalling.^13^ Furthermore, RASAL3 is a T cell‐specific RAS‐GTP active protein that negatively regulates TCR‐induced RAS/MAPK pathway activation.^15^ RASLA3‐deficient mice had fewer naive T cells because RASAL3 is necessary for naive T cell survival.^40^

Recently, previous studies have revealed that TILs play important roles in tumour microenvironment and affect tumour progression, as well as are associated with prognosis.^41^, ^42^, ^43^, ^44^, ^45^ T lymphocytes are divided into the following subtypes: CD4 + T helper lymphocytes (Th), CD45RO+ memory T cells (Tm), CD8+ cytotoxic T lymphocytes (CTL), FOXP3+ regulatory cells (Tregs), etc., which are classified according to cell surface markers. High CD8+ T cell levels in the tumour stroma and tumour nest were linked to enhanced overall survival in lung cancer patients.^20^, ^46^

In this study, we used CIBERSORT algorithm to explore the correlation between RASAL3 and immune cell infiltration. CIBERSORT results revealed that RASAL3 expression was significantly associated with CD8+ T lymphocyte infiltration. In addition, the correlations between RASAL3 and genetic markers of immune cells indicated that RASAL3 exhibited a moderate association with CD8+ T cells. Furthermore, we used TMA‐based IHC to confirm a positive association between RASAL3 expression level and CD8+ tumour‐associated T lymphocyte infiltration. Our results indicated that RASAL3 expression may be related to CD8+ T lymphocyte infiltration in LUAD. RASAL3 expression was linked to prognosis and CD8+ tumour‐associated T lymphocyte infiltration in LUAD, indicating its potential as a predictive biomarker and immune‐related therapeutic target for LUAD.

We must consider the limitations of the present analysis. First, we found that RASAL3 might be linked to LUAD progression and CD8+ T lymphocyte infiltration, but the mechanism is unclear. Accordingly, we will create silenced‐RASAL3 LUAD cell lines and overexpressed RASAL3 LUAD cell lines for future research, to study RASAL3 function in T lymphocyte polarization and differentiation, and whether RASAL3 expression affects the secretion of cytokines and/or metabolites implicated in T lymphocyte recruitment. In addition, we used only 82 LUAD tissues to validate the RASAL3 prognostic effect and RASAL3 with CD8+ T lymphocyte infiltration correlation. In future research, we will increase the sample size to validate these findings and explore whether RASAL3 affects other immune cell infiltration. The RASAL3 molecular mechanism in LUAD requires further investigation.

In summary, RASAL3 high expression was linked to a better LUAD prognosis and increased CD8+ T lymphocyte immune infiltration levels in LUAD. RASAL3 might have a significant function in CD8+ T lymphocyte infiltration and may be a biomarker for the prognosis of LUAD patients.

## AUTHOR CONTRIBUTIONS

**Mei Liang:** Conceptualization (lead); data curation (lead); formal analysis (lead); investigation (lead); methodology (lead); resources (lead); software (lead); supervision (lead); validation (lead); visualization (equal); writing – original draft (lead); writing – review and editing (lead). **Xiangzhi Meng:** Conceptualization (supporting); data curation (equal); project administration (equal); resources (supporting); software (supporting). **Boxuan Zhou:** Investigation (equal); methodology (equal); resources (supporting); validation (supporting). **Yushun Gao:** Conceptualization (equal); data curation (equal); formal analysis (equal); funding acquisition (lead); investigation (equal); methodology (equal); project administration (equal); resources (equal); supervision (lead); validation (equal); visualization (equal); writing – review and editing (equal).

## FUNDING INFORMATION

This work was supported by the National Key R&D Program of China (Grant Nos. 2020YFE02022200).

## CONFLICT OF INTEREST

The authors declare that the research was conducted in the absence of any commercial or financial relationships that could be construed as a potential conflict of interest.

## Data Availability

Data availability statement ;The publicly available data sets were analyzed in this study. The authors confirm that these data can be found here: http://www.abren.net/PrognoScan/,http://xena.ucsc.edu/, https://gdc.cancer.gov/about‐data/publications/panimmune,http://timer.comp‐genomics.org/.The TMA‐based IHC results or any other data presented in this study are available from the corresponding author upon reasonable request.
